# Industry Payments to Orthopedic Surgeons Among All Subspecialties: An Analysis of the Open Payments Database From 2014 to 2019

**DOI:** 10.7759/cureus.54981

**Published:** 2024-02-26

**Authors:** John M Tarazi, Nicholas Frane, Alain E Sherman, Peter B White, Matthew Partan, Emma K Humphrey, Adam Bitterman

**Affiliations:** 1 Department of Orthopedic Surgery, Donald and Barbara Zucker School of Medicine at Hofstra/Northwell, Hempstead, USA; 2 Department of Orthopedic Surgery, Northwell Health—Huntington Hospital, Huntington, USA; 3 Department of Orthopedic Surgery, The Center for Orthopedic Research and Education (CORE) Institute, Phoenix, USA; 4 Department of Orthopedic Surgery, Northwell Health—Lenox Hill Hospital, New York, USA; 5 Department of Orthopedics, Ohio University Heritage College of Osteopathic Medicine—Cleveland, Warrensville Heights, USA

**Keywords:** open payments database, payments from industry, orthopedic surgery, centers for medicare and medicaid services, industry payment

## Abstract

Introduction

Since the passage of the Physician Payments Sunshine Act in 2010, the Centers for Medicare and Medicaid Services (CMS) started the National Physician Payment Transparency Program and Open Payments Database (OPD), which allowed for public access to financial disclosures between physicians and industry. Although orthopedic surgeons receive the highest average payments when compared to other specialties, there has been limited data evaluating these payments among the different orthopedic subspecialties. The purpose of this study was to analyze all industry payments made across all subspecialties among orthopedic surgeons.

Methods

A retrospective review of the CMS OPD was performed to identify all industry payments made by drug and medical device companies to orthopedic surgeons (N = 28,475) between January 1, 2014, and December 31, 2019. Descriptive statistics were calculated for the number, individual value, and total value of industry payments, stratified by payment type and orthopedic subspecialty.

Results

A total of 1,048,573 payments (approximately $1.6 billion) were made to orthopedic surgeons between 2014 and 2019. The average orthopedic surgeon received 6.14 payments per year (SD = 29.39), with a mean individual payment amount of $1,542.32. Royalties or licensing comprised the greatest proportion of open payments, followed by consulting fees. Adult reconstruction (M = $225,131.10) and spine (M = $197,404.74) received significantly greater total payments when compared to all other subspecialties (all p-values ≤ 0.001). Differences in total payments made to trauma (M = $73,789.65), sports medicine (M = $60,988.09), foot and ankle (M = $45,007.45), pediatric orthopaedics (M = $35,898.54), general orthopaedics (M = $28,405.81), and hand (M = $14,027.76) were all found to be statistically equivalent (all p­-values > 0.20).

Discussion

Increased collaboration between physicians and industry has resulted in the rapid advancement of innovation that can have sizeable financial implications among orthopedic surgeons. There exists significant heterogeneity in open payments made to orthopedic surgeons when stratified by subspecialty. Adult reconstructive and spine surgeons were the most compensated whereas hand and general orthopaedic surgeons received the least.

## Introduction

In recent years, increased collaboration between physicians and industry has resulted in the rapid advancement of innovation within the field of orthopedic surgery. Although the 20th century has witnessed continued innovation in orthopedic surgery, an argument can be made that some of the largest advancements have been observed in the last twenty years alone [1]. Novel medical technologies, implants, biologic and regenerative therapies have transformed the specialty for the benefit of millions of patients [2-6]. However, as these two parties continue to innovate, there is an ethical responsibility to ensure that the financial agreements behind these partnerships do not adversely impact patient care [7-10]. In order to monitor the relationships between physicians and industry and increase public transparency, the Physician Payments Sunshine Act (PPSA) was launched in 2010 [11]. For the first time, this legislation required all drug, device, biological, and medical supply companies to report any monetary transfer greater than $10 (U.S. Dollars) to physicians to the U.S. Centers for Medicare and Medicaid Services (CMS) [11]. Subsequently, CMS initiated the National Physician Payment Transparency Program and Open Payments Database (OPD) which allowed for public access to all financial disclosures exchanged between physicians and industry [12,13]. Although some critics viewed the OPD as an attempt to deter physicians from accepting payments, others considered it as an opportunity to bolster trust within the medical profession [13-16].

With a growth of 8.7% in industry payments occurring between 2014 and 2019, orthopedic surgeons are among the highest compensated in industry payments, and it is imperative to better understand this dynamic [17]. However, given the various subspecialties of orthopedic surgery, there are noticeable comparisons that might be made. Therefore, the purpose of this study was to analyze trends in industry payments made to orthopedic surgeons between 2014 to 2019. We sought to examine different types of industry payments as well as differences between the various orthopedic subspecialties. Within the OPD, payments include several subdivisions that include total general payments that were calculated as the sum of all subcategories and were recorded for each year. In doing so, as a consequence of the PPSA, the authors examine the (1) individual industry payments and (2) total industry payments among each of the orthopedic subspecialties. 

## Materials and methods

A retrospective review of the CMS OPD was performed to identify all industry payments made by drug and medical device companies to orthopedic surgeons between January 1, 2014, and December 31, 2019 [18]. The OPD is available in the public domain and includes all payments/transfers made to licensed physicians across all medical disciplines who receive greater than or equal to $100 annually. Orthopedic surgeons who received total annual payments of less than $100 or no open payments between 2014 and 2019 are excluded from the OPD. Each payment is listed in the database with an associated reason: faculty or speaker fees; consulting fees; ownership or investment interests; education; entertainment, food, and beverage; gifts; grants; honoraria; royalty or licensing; and travel and lodging [19]. Payments are compiled annually and released as part of datasets. To date, the OPD has released seven datasets beginning in the latter half of 2013. All complete annual datasets from 2014 through 2019 were queried. In concordance with other similar studies, the 2013 dataset was excluded due to incomplete data [2,20-22]. Given that the OPD is comprised of publicly available data and does not contain protected health information, this study was deemed to be exempt from Institutional Review Board (IRB) review.

The sample for this study was comprised of all allopathic and osteopathic physicians specializing in orthopedic surgery listed in the OPD (N = 28,475). Stratified by orthopedic subspecialty, the sample consisted of general orthopedic surgeons (n = 15,510; 54.5%), sports medicine surgeons (n: 3,808; 13.4%), orthopedic hand surgeons (n: 2,662; 9.3%), orthopedic spine surgeons (n: 2,375; 8.3%), adult reconstructive orthopedic surgery (n: 1,297; 4.6%), orthopedic foot and ankle surgeons (n: 1,219; 4.3%), orthopedic trauma surgeons (n: 892; 3.1%), and pediatric orthopedic surgeons (n: 712; 2.5%). The OPD does not include orthopedic oncologists and shoulder and elbow orthopedic surgeons in their database, therefore they were not included in our study.

All data were analyzed using the Statistical Package for the Social Sciences (SPSS) version 23.0 (IBM Corp., Armonk, NY). Descriptive statistics (i.e., mean, median, standard deviation, range) and frequencies were calculated for the number and monetary value of individual industry payments. In addition, these values were calculated for total industry payments by orthopedic surgeons. Trends in payment data over time were analyzed by linear regression modeling. One-way analyses of variance (ANOVAs) with Tukey honestly significant difference (HSD) post-hoc tests were performed to compare individual and total payment data by orthopedic subspecialty. The criterion for statistical significance was set at α = 0.05 for all inferential tests.

## Results

A total of 1,048,573 payments; totaling $1,617,234,197.17; made to 28,475 orthopedic surgeons were identified between 2014 and 2019. The average orthopedic surgeon received 6.14 payments per year (SD = 29.39, Range 1, 327). Mean payment amounts did not change significantly over time, r = 0.0003, p = 0.76 (Table 1). The greatest number of open payments was made to general orthopedic surgeons (n = 432,830; 41.3%), followed by orthopedic spine surgeons (n = 192,361; 18.3%) and orthopedic surgeons specializing in sports medicine (n = 147,194; 14.4%) (Table 2). Pediatric orthopedic surgeons received the lowest number of payments (n = 16,293; 1.6%).

**Table 1:** Individual Industry Payments Over Time

Year	N	Mean ($)	SD ($)	Total ($)
2014	305,681	1,498.73	25,569.37	458,134,474.77
2015	304,252	1,543.23	65,935.75	469,529,283.55
2016	99,150	1,765.40	26,902.34	175,039,592.11
2017	111,218	1,657.52	33,082.99	184,345,894.06
2018	107,703	1,507.70	20,143.23	162,383,292.51
2019	120,569	1,391.75	19,083.63	167,801,660.17
Total	1,048,573	1,542.32	41,474.59	1,617,234,197.17

**Table 2:** Individual Industry Payments by Orthopedic Subspecialty

Subspecialty	N	%	Mean ($)	SD ($)	Median ($)	Minimum ($)	Maximum ($)
Adult Reconstruction	94,265		2,846.45	30,573.03	81.77	0.03	4,784,208.28
		9					
Spine	192,361	18.3	2,171.95	80,278.23	50.59	0	33,579,000.00
General	432,830	41.3	1,383.28	28,550.47	36.49	0	6,378,733.80
Sports	147,194	14	1,239.02	27,485.79	42.15	0.01	2,237,683.76
Foot & Ankle	53,280	5.1	1,003.28	6,055.23	60.19	0	481,621.81
Trauma	44,997	4.3	953.14	11,271.95	72.71	0.26	1,749,687.40
Pediatrics	16,293	1.6	724.45	5,429.83	64.11	0.1	274,098.32
Hand	67,353	6.4	621.63	7,081.74	36.52	0	1,104,464.29

**Figure 1:**
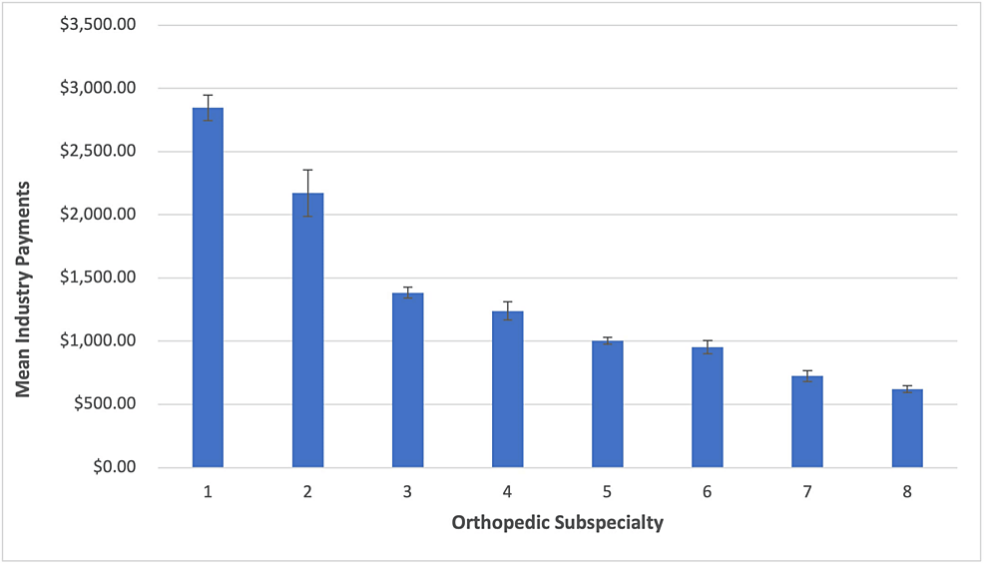
Mean Individual Industry Payments by Orthopedic Subspecialty

The mean of individual payments made to orthopedic surgeons was $1,542.32 (SD = $41,474.58), with a median of $44.77 and a range of [$0.00, $33,579,000.00]. A one-way ANOVA revealed a highly statistically significant main effect of orthopedic subspecialty on mean individual payment amount, F = 29.92, p < 0.001 (Figure 1). The largest individual payments were made to adult reconstructive orthopedic surgeons (M = $2,846.45; SD = $30,573.03), followed by orthopedic spine surgeons (M = $2,171.95; SD = $80,278.23). By contrast, orthopedic hand surgeons (M = $621.63, SD = $7,081.74) and pediatric orthopedic surgeons (M = $724.45, SD = $5,429.83) received individual payments of the smallest monetary value.

Tukey HSD post-hoc tests revealed that adult reconstructive orthopedic surgeons received significantly greater mean individual payments when compared to all other subspecialties, including orthopedic spine surgery (all p-values ≤ 0.001) (Table 3). Similarly, the mean of individual payments made to orthopedic spine surgeons was significantly greater than those of all other subspecialties, with the exception of adult reconstruction (all p-values ≤ 0.001). General orthopedic surgeons received significantly greater mean individual payments when compared to orthopedic hand surgeons (p < 0.001). However, overall differences in mean individual payments between general orthopedic surgeons, foot and ankle surgeons (p = 0.49), trauma surgeons (p = 0.42), pediatric surgeons (p = 0.49), and sports medicine surgeons (p = 0.95) were found to be statistically equivalent. Lastly, orthopedic hand surgeons received significantly smaller mean individual payments when compared to adult reconstruction surgeons (p < 0.001), orthopedic spine surgeons (p < 0.001), general orthopedic surgeons (p < 0.001), and sports medicine surgeons (p = 0.03), but not foot and ankle surgeons (p = 0.76), trauma surgeons (p = 0.89), or pediatric surgeons (p > 0.999).

**Table 3:** Post-hoc Analysis of Mean Individual Payments for Orthopedic Subspecialties

Index Subspecialty	Comparison Subspecialty	Mean Difference ($)	p-Value
Adult Reconstruction	Hand	2224.82	< 0.001
	Pediatrics	2122	< 0.001
	Trauma	1893.3	< 0.001
	Foot and Ankle	1843.16	< 0.001
	Sports	1607.43	< 0.001
	General	1463.17	< 0.001
	Spine	674.5	0.001
Spine	Hand	1550.32	< 0.001
	Pediatrics	1447.5	0.001
	Trauma	1218.81	< 0.001
	Foot and Ankle	1168.67	< 0.001
	Sports	932.93	< 0.001
	General	788.67	< 0.001
	Adult Reconstruction	-674.5	0.001
General	Hand	761.65	< 0.001
	Pediatrics	658.83	0.488
	Trauma	430.13	0.419
	Foot and Ankle	380	0.485
	Sports	144.25	0.945
	Spine	-788.67	< 0.001
	Adult Reconstruction	-1463.17	< 0.001
Hand	Peds	-102.82	> 0.999
	Trauma	-331.52	0.894
	Foot and Ankle	-381.65	0.758
	Sports	-617.39	0.03
	General	-761.65	< 0.001
	Spine	-1550.32	< 0.001
	Adult Reconstruction	-2224.82	< 0.001

**Figure 2:**
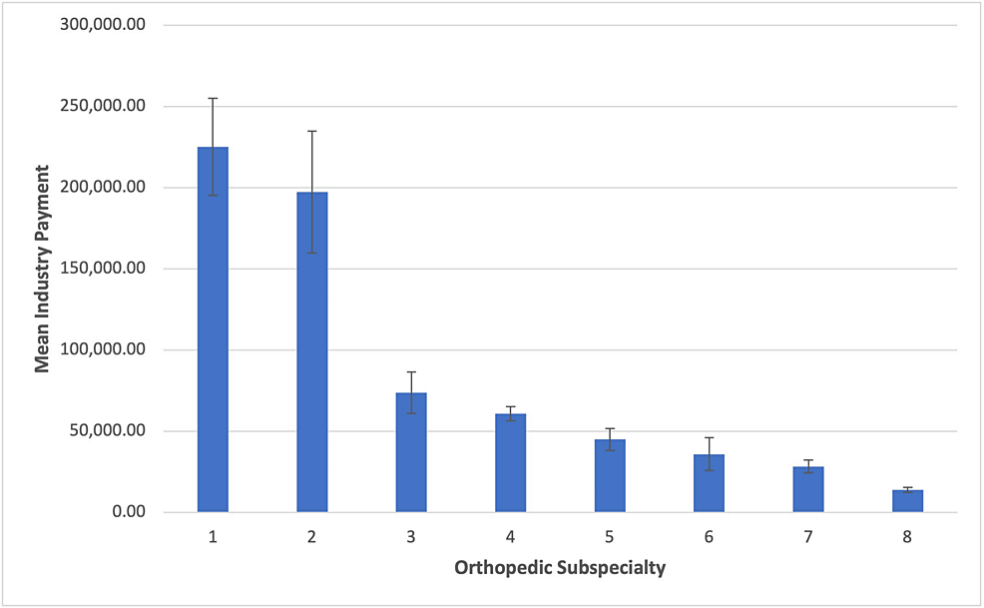
Mean Total Industry Payments by Orthopedic Subspecialty

The mean of sum total payments made to orthopedic surgeons over the six-year period was $56,794.88 (SD = $756,549.48), with a median of $1,344.56 and range of [$1.56, $80,300,759.80] (Table 4). A one-way ANOVA revealed a highly statistically significant main effect of orthopedic subspecialty on the mean total payment amount, F = 25.58, p < 0.001 (Figure 2). On average, adult reconstructive orthopedic surgeons received the greatest total payments (M = $225,131.10; SD = $1,080,655.95), followed by orthopedic spine surgeons (M = $197,404.74; SD = $1,828,044.60). By contrast, orthopedic hand surgeons (M = $14,027.76, SD = $81,302.80) and general orthopedic surgeons (M = $28,405.81, SD = $493,320.39) received total payments of the smallest monetary value among all subspecialties.

**Table 4:** Total Industry Payments by Orthopedic Subspecialty

Subspecialty	N	Mean ($)	SD ($)	Median ($)	Minimum ($)	Maximum ($)
Adult Reconstruction	1,297	225,131.10	1,080,655.95	5,196.31	6.46	17,447,689.40
Spine	2,375	197,404.74	1,828,044.60	7,008.61	1.56	80,300,759.80
Trauma	892	73,789.65	377,744.96	5,104.80	11.73	7,600,732.44
Sports	3,808	60,988.09	269,785.33	3,952.53	4.19	45,064,907.60
Foot and Ankle	1,219	45,007.45	236,622.49	2,043.54	2.79	4,825,760.40
Pediatrics	712	35,898.54	269,785.33	1,868.42	10.81	4,689,584.55
General	15,510	28,405.81	493,320.39	621.14	1.56	52,684,638.60
Hand	2,662	14,027.76	81,302.80	1,484.90	1.9	2,396,954.94
Total	28,475	56,794.88	843,691.09	1,344.56	1.56	80,300,759.80

On average, adult reconstructive orthopedic surgeons received the greatest total payments (M = $225,131.10; SD = $1,080,655.95), followed by orthopedic spine surgeons (M = $197,404.74; SD = $1,828,044.60). By contrast, orthopedic hand surgeons (M = $14,027.76, SD = $81,302.80) and general orthopedic surgeons (M = $28,405.81, SD = $493,320.39) received total payments of the smallest monetary value among all subspecialties.

Tukey HSD post-hoc tests revealed that adult reconstructive orthopedic surgeons and orthopedic spine surgeons received significantly greater mean total payments when compared to all other subspecialties (all p-values ≤ 0.001) (Table 5). However, overall mean total payments between adult reconstruction and orthopedic spine surgeons were not found to be significantly different (p = 0.94). Differences in mean total payments made to orthopedic trauma surgeons, sports medicine surgeons, foot and ankle surgeons, pediatric surgeons, general orthopedic surgeons, and hand surgeons were all found to be statistically equivalent (all p­-values > 0.20).

**Table 5:** Post-hoc Analysis of Mean Total Payments for Orthopedic Subspecialties

Index Subspecialty	Comparison Subspecialty	Mean Difference ($)	p-Value
Adult Reconstruction	General	196,725.29	< 0.001
	Foot and Ankle	180,123.65	< 0.001
	Hand	211,103.34	< 0.001
	Spine	27,726.36	0.936
	Trauma	151,341.44	< 0.001
	Pediatrics	189,232.56	< 0.001
	Sports	164,143.00	< 0.001
Spine	General	168,998.93	< 0.001
	Adult Reconstruction	-27,726.36	0.964
	Foot and Ankle	152,397.29	< 0.001
	Hand	183,376.98	< 0.001
	Trauma	123,615.08	0.001
	Pediatrics	161,506.20	< 0.001
	Sports	136,416.64	< 0.001
Trauma	General	45,383.84	0.656
	Adult Reconstruction	-151,341.44	< 0.001
	Foot and Ankle	28,782.21	0.989
	Hand	59,761.90	0.449
	Spine	-123,615.08	0.001
	Pediatrics	37,891.11	0.975
	Sports	12,801.56	> 0.999

**Figure 3:**
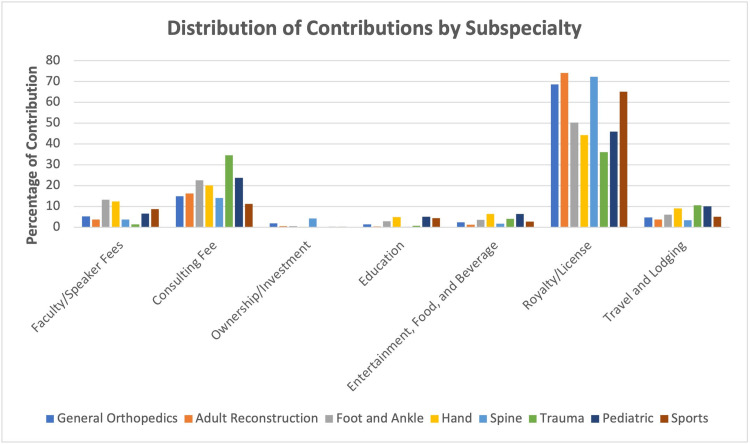
Distribution of Contributions by Payment Type

There was a statistically significant interaction between payment type and orthopedic subspecialty, F = 42.05, p < 0.001. Royalties or licensing comprised the greatest proportion of open payments for all orthopedic subspecialties, followed by consulting fees (Figure 3). The majority of payments were made to adult reconstructive surgeons (74.19%), orthopedic spine surgeons (72.34%), general orthopedic surgeons (68.68%), sports medicine surgeons (65.08%), and foot and ankle surgeons (50.21%) came from royalties or licensing. Ownership or investment interests; education; and entertainment, food, and beverage contributed to less than 10% of open payments made to all subspecialties.

## Discussion

Conflicts of interest in orthopedic surgery have been a topic of considerable discussion over the past two decades [23-25]. With the highest per-physician general payment value among medical and surgical specialties, orthopedic surgeons receive the most substantial industry payments [26-29]. Despite many patients suggesting that surgeon-industry relationships have the potential to provide superior patient care, many also believe that all financial relationships and conflicts of interest should be disclosed to patients [20,30,31]. Given the self-reporting inconsistencies, the PPSA was created in order to resolve those issues and implement more transparency and consistency; however, unlike other specialties, which have seen fractured relationships between physicians and industry, the relationship between orthopedic surgeons and industries has only strengthened since passage of the act [2,21,32-34]. To our knowledge, this is the first study to examine individual and total industry payments among all orthopedic subspecialties with direct comparisons and contributions by payment type. In our study, a total of 1,048,573 payments (approximately $1.6 billion) were made to orthopedic surgeons between 2014 and 2019. The average orthopedic surgeon received 6.14 payments per year, with a mean individual payment amount of $1,542.32. Royalties or licensing comprised the greatest proportion of open payments, followed by consulting fees. Adult reconstruction and spine received significantly greater total payments when compared to all other subspecialties. Differences in total payments made to trauma, sports medicine, foot and ankle, pediatric orthopedics, general orthopedics, and hand were all found to be statistically equivalent.

In a study that examined CMS OPD payments between 2013 and 2017, Robin et al. concluded that orthopedic spine surgeons are the highest compensated, while sports medicine orthopedic surgeons receive higher total mean payment amounts [35]. This study captured the 25 highest-compensated orthopedic surgeons during this period and filtered to include only 917 physicians, which represented 347 unique orthopedic surgeons after removing duplicates. Although a much smaller study, Robin et al. focused on the top earnings within each orthopedic subspecialty [35]. They also concluded that more than 20% of the total sum of earnings was earned by just four physicians, suggesting that the gross sum of reimbursement is skewed by a select few. Furthermore, this study determined a significant relationship between the company and both mean payment amount and frequency of payments, as well as royalty and licensing payments serving as the main mechanism of payment among payees. In our present study, similar findings were reported; however, with a much larger sample size and longer period, our study further itemized the contributions among each payment type. By appreciating this impact, we can better explain the increased avenues for industry-physician relationships. 

The financial scope of the interactions made between healthcare companies and physicians can range from the cost of meals and travel to royalty and licensing payments, to consulting and speaker fees [17]. In a 2015 study, Cvetanovich et al. reported that orthopedic surgeons received more industry payments than neurological surgeons, urologists, plastic surgeons, and otolaryngologists [9]. Furthermore, royalties and licensing fees, which were received by 1.7% of orthopedic surgeons, accounted for 69.5% of the total monetary value of payments to orthopedic surgeons [9]. Over our six-year study, the sum total of individual industry payments over time amounted to over $1.6 billion (see Table 1). One of the primary reasons for this is the clinical and technical expertise that orthopedic surgeons can offer medical device companies that are aiming to develop novel devices [29]. Moreover, previous studies have concluded that orthopedic surgeons hold the largest proportion of medical device patents compared to other medical specialties [36]. However, of these sub-payment categories, royalties and licensing agreements have been demonstrated to be responsible for the major contributions of industry payments [9,20,21,37].

Whether it is the digital modeling of robotic navigation software or the clinical efficiency of hip or knee joint replacements, some may argue that joint arthroplasty has been one of the most innovative subspecialties within orthopedic surgery [38,39]. According to a recent research report in 2020, the global orthopedic implants market size is expected to exceed $6.89 billion by the end of 2026 [40]. With the recent paradigm shift from conventional surgical procedures to cutting-edge implants, adult reconstructive surgery, especially, will continue to find prominence across the geriatric population as continual investments by key markets do not appear to be slowing down. Additionally, our study demonstrated that orthopedic spine surgeons have the second-highest contributions from royalties and licensing agreements. Similar to the recent reports on joint reconstruction, with the growing incidence of spinal cord injuries, degenerative processes, and surgeries, the market for spine implants such as pedicle screw systems, lumbar disc replacements, and vertebral compression devices are estimated to approach a combined $7 billion by 2027 [40,41]. These findings therefore highlight the close, symbiotic relationship between industry and surgeon being implemented to enhance patient care and advancements in their respective fields. 

In a 2015 study, Lieber et al. assessed 2,555 orthopedic surgeons who received an industry consulting fee and demonstrated a statistically significant relationship between high industry compensation and greater scholarly impact [29]. At the time of this study, however, Lieber et al. reported that fellowship-trained surgeons in adult reconstruction and foot and ankle had the strongest ties to research productivity [29]. Since that time, our study demonstrated that orthopedic trauma surgeons are the highest recipients of this sub-payment type. Although we reported that fellowship-trained surgeons in foot and ankle remained as one of three subspecialties that received the most payments in consulting fees, it serves to wonder if research productivity still holds true among orthopedic trauma surgeons in 2022.

The present study is not without limitations. The study contains some limitations aside from its retrospective nature. First, the results of our analysis are limited by the accuracy and inclusiveness of OPD data submitted to CMS. As such, inconsistent reporting has been mostly reportedly seen among general orthopedic surgeons [22,42]. Second, since the data are submitted by industry, there is a risk for selection and reporting bias as some surgeons are not appropriately labeled based on subspecialty. This can be seen with the absence of orthopedic oncologists and shoulder and elbow orthopedic surgeons, as they are not included in the OPD. Third, the existing database has several inherent limitations including the validity of the data and the variables that are recorded. As mentioned by Callaghan and Liu, the database captures payment type and amount but fails to account for important variables of true value ie. legal and ethical definitions not provided within the database [20,43]. Including other variables such as the value of payments with respect to companies (i.e., total sales, research and development, marketing, etc.) would be of value as well [20]. Future studies should further define the true value of compensation and look to incorporate other sets of variables. Fourth, discrepancies in payments between genders among orthopedic surgeons were not included in this analysis. Ray et al. demonstrated that 99.6% of payments were being made to male physicians [44]. Although historically a male-dominated field, the increasing presence of women in orthopedic surgery makes addressing this concern of inequality more critical [45-47]. Fifth, the dataset only includes published data; therefore, it fails to account for unidentified records; or records that are withheld due to manufacture requests, publication delays, unresolved physician disputes; or failed submissions for covered recipients [48]. Despite these limitations, the OPD is currently the largest and most robust dataset of physician-industry relationships available. Future studies should further define the true value of compensation and look to incorporate other sets of variables. Although additional revisions should be implemented to improve the accuracy of reporting, the authors believe that the data sample is large enough to satisfy the goals of this study.

## Conclusions

Greater awareness of public reporting from the Open Payments Database is imperative with the continued role of physician involvement in product development and industry success. In summary, the largest individual payments are being made to adult reconstructive and orthopaedic spine surgeons who receive significantly greater mean individual payments when compared to all other subspecialties. The mean of total payments made to orthopedic surgeons was $56,794.88, with adult reconstructive and orthopedic spine surgeons receiving the greatest total payments and orthopedic hand and general orthopedic surgeons receiving the least.
